# Association between muscle strength and mass and bone mineral density in the US general population: data from NHANES 1999–2002

**DOI:** 10.1186/s13018-023-03877-4

**Published:** 2023-06-01

**Authors:** Huawei Han, Shuai Chen, Xinzhe Wang, Jie Jin, Xianghui Li, Zhiwei Li

**Affiliations:** 1 Department of Orthopaedics, The Second Affiliated Hospital of Nanjing University of Chinese Medicine, No.23, Nanhu Road, Jianye District, Nanjing, 210017 Jiangsu Province People’s Republic of China; 2 Department of Gynecology, Jiangsu Province Hospital of Chinese Medicine, Affiliated Hospital of Nanjing University of Chinese Medicine, Nanjing, People’s Republic of China

**Keywords:** Muscle strength, Muscle mass, Bone mineral density, Osteoporosis

## Abstract

**Purpose:**

It is known that muscle strength and muscle mass play a crucial role in maintaining bone mineral density (BMD). Despite this, there are uncertainties about how muscle mass, lower extremity muscular strength, and BMD are related. We examined the impact of lower extremity muscle strength and mass on BMD in the general American population using cross-sectional analysis.

**Methods:**

In the study, we extracted 2165 individuals from the National Health and Nutrition Examination Survey 1999–2002. Multivariate logistic regression models were used to examine the association between muscle strength, muscle mass, and BMD. Fitted smoothing curves and generalized additive models were also performed. To ensure data stability and avoid confounding factors, subgroup analysis was also conducted on gender and race/ethnicity.

**Results:**

After full adjustment for potential confounders, significant positive associations were detected between peak force (PF) [0.167 (0.084, 0.249) *P* < 0.001], appendicular skeletal muscle index (ASMI) [0.029 (0.022, 0.036) *P* < 0.001], and lumbar spine BMD. A positive correlation was also found between PF, ASMI, and pelvis and total BMD. Following stratification by gender and race/ethnicity, our analyses illustrated a significant correlation between PF and lumbar spine BMD in both men [0.232 (0.130, 0.333) *P* < 0.001] and women [0.281 (0.142, 0.420) *P* < 0.001]. This was also seen in non-Hispanic white [0.178 (0.068, 0.288)* P* = 0.002], but not in non-Hispanic black, Mexican American and other race–ethnicity. Additionally, there was a positive link between ASMI and BMD in both genders in non-Hispanic whites, and non-Hispanic blacks, but not in any other racial group.

**Conclusion:**

PF and ASMI were positively associated with BMD in American adults. In the future, the findings reported here may have profound implications for public health in terms of osteopenia and osteoporosis prevention, early diagnosis, and treatment.

## Introduction

Described as a disease of the bones, osteopenia and osteoporosis result in reduced bone mass and degeneration of bone tissue structure, which increases susceptibility to bone fragility and fracture [1]. By 2030, there will be 13.5 million people with osteoporosis in the USA, up from 10.2 million in 2010, and projections estimate that over 47 million Americans will be afflicted with osteopenia in 2020 [2–5]. Osteoporotic fracture is a serious clinical complication of osteoporosis, which is responsible for more than 1.5 million fractures annually [6]. Approximately two million dollars was spent on osteoporosis-related unintentional fractures in 2005, and the costs continue to rise [7]. Due to the growing morbidity and mortality, and expense of healthcare, osteoporosis has emerged as a significant public health issue.

A number of risk factors contribute to osteopenia and osteoporosis, including hormonal factors, low peak bone mass, smoking, low physical activity, race/ethnicity, and low strength and muscle. In assessing the risk of low bone mass, these factors should all be considered. Among them, muscle strength, muscle mass, and BMD have triggered extensive research due to the interdependence of the skeletal and muscular systems. However, the relationship between them is unclear, and the outcomes associated with this relationship are controversial. Ahedi et al. [8] discovered that hip muscle cross-sectional area and muscle strength were positively correlated with hip BMD. Similarly, Zhou et al. [9] also explored the connection between muscle strength and BMD and confirmed that the decrease in muscle strength was positively correlated with the decrease in BMD. Furthermore, in both men and women, a cross-sectional study demonstrated a link between BMD and muscle mass [10]. However, some researchers reported no relationship between muscular strength and BMD. According to a cross-sectional investigation, the isokinetic strength of hip muscles may not contribute to the BMD of the proximal femur [11]. Similarly, a cross-sectional study of 58 women (aged 62.1 ± 4.8 years) indicated an association between body fat mass and BMD of the proximal femur but not in lean body mass or appendicular lean mass (ALM) [12]. The emergence of such contradictory conclusions may be due to different study designs, different muscle measurement instruments, and problems with sample sizes between studies. Therefore, we used data from the larger data of NHANES and a more established method of muscle measurement.

Muscle strength is a measure of how a muscle can exert maximum strength. Presently, most of the research in the area of muscle strength is on grip strength. It mainly focuses on the hand and forearm measurement of power. Nevertheless, the main anatomical area for muscle function measurement is in the lower body, and these muscles are essential for daily activities. Additionally, lower limb strength loss is the biggest risk factor leading to falls and other injuries and disabilities [13, 14]. Thus, it will be of more clinical significance to explore the correlation between lower limb muscle strength and BMD to intervene, protect, and prevent osteoporosis. We evaluated muscle mass by calculating the Appendicular Skeletal Muscle Mass Index (ASMI) [15], and as appendicular muscle mass is less confounded by noncontractile lean body mass differences, we selected it over total lean body mass for our analysis. Therefore, we aimed to explore the feasibility of using the lower extremity muscle peak force (PF) value as an indicator of muscle strength and ASMI to predict BMD based on cross-sectional data from the 1999–2002 NHANES.

## Materials and methods

### Study population

All data in this study were obtained from the National Health and Nutrition Examination Survey (NHANES), a major, ongoing cross-sectional survey conducted by the Centers for Disease Control and Prevention that has released data in two year increments since 1999. The NHANES participants signed informed consent prior to the implementation of the NHANES protocols approved by the National Center for Health Statistics Research Ethics Review Board [16].

**Fig. 1:**
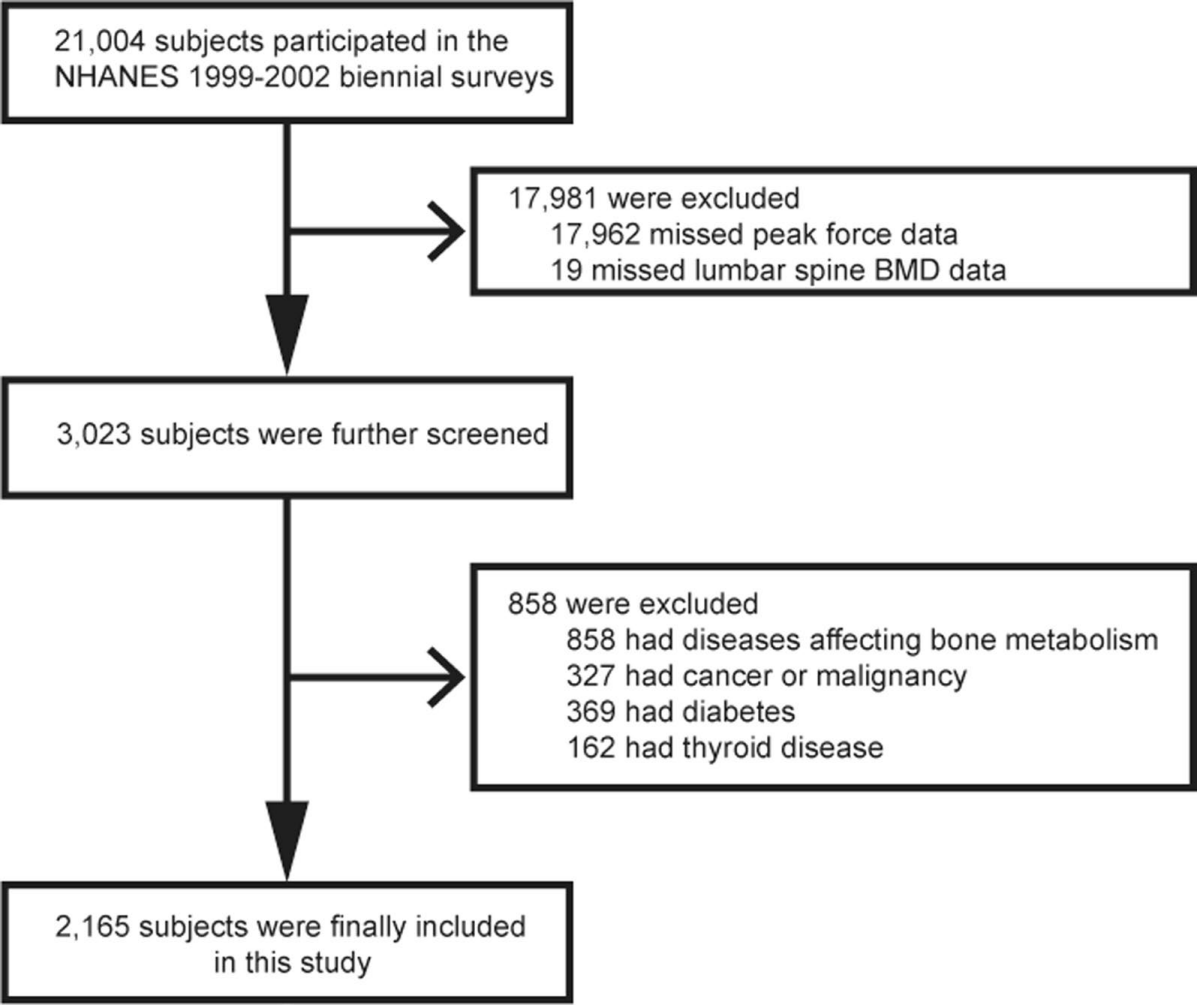
Study flowchart. NHANES, National Health and Nutrition Examination Survey; BMD, body mineral density

The NHANES datasets were utilized for this investigation from 1999 to 2002. In total, there were 21,004 participants who completed nutrition and health condition questionnaires, as well as health examinations. Consequently, 2165 participants were included in this study due to the following: (1) there were no peak force data (*n* = 17,962); (2) no Lumbar spine BMD data (*n* = 19); and (3) patients with cancer, thyroid disease, or diabetes (*n* = 858) (Fig. 1).

### Definitions of muscle strength and mass

#### Muscle strength

It is reported that Kinetic Communicator MP isokinetic dynamometers (Chaptex Corp., Chattanooga, TN) were used by NHANES to measure voluntary peak knee extensor strength. Six measurements of muscle strength were taken at 60 degrees per second on the right quadriceps muscle [17, 18]. Analyses were excluded from those individuals with abnormal force–velocity limits (under 55 degrees/second or above 65 degrees/s) [19].

This portion of the test was used to learn movements and warm up, so individuals were instructed not to exert their maximal effort during the first three trials. Muscle strength was measured during the last three trials with maximum effort. A maximum peak force value was selected if they had completed 4–6 trials [20].

#### Muscle mass

Using a DXA QDR-4500 Hologic scanner, appendicular skeletal muscle mass (ASM) was determined as the combined muscle mass of the legs and arms [21]. It was determined that all muscle tissue, excluding fat and bone mass, was skeletal muscle, while ASM was defined as all lean soft tissue in the limbs [22]. In our study, ASMI was used to quantify muscle mass. The ASMI was calculated with ASM (kg) and height (m), where ASMI = ASM/height^2^ [15].

### BMD measurement

In this study, BMD was measured by dual-energy X-ray absorption (DXA) with a Hologic QDR 4500A fan beam densitometer (Hologic Inc., Bedford, MA, USA) [23]. In order to assess fragility-fracture risk, this screening tool is commonly used internationally. We used radiologic technologists who are certified and trained to administer DXA examinations. In the survey, all participants aged 8 and older were eligible to receive a DXA scan. DXA is not suitable for pregnant women, individuals weighing over 300 pounds, or those who have taken radiographic contrast material in the past 7 days [22].

### Other covariates

The covariates are demographic data, examination data, and questionnaire data. Demographic data included age (years, range 50–85, average: 64), gender (male and female), race–ethnicity, level of education (less than high school graduate, some college, missing), annual family income ($0–19,999, Over $20,000, missing), and marital status (separated/divorced/widowed/never married, married/living with partner, missing/refused/don’t know). Examination data included weight (kg), height (m), body mass index (BMI, kg/m^2^). Additionally, questionnaire data included information on smoking behavior (yes/no), arthritis (yes/no), physical activity, alcohol consumption (yes/no), and hypertension. Phosphorus (mg/dL), energy (kcal/day), total fat (g/day), and caffeine (mg/day) intake were considered potential confounders [24–27]. The level of physical activity done in the past 30 days was defined as moderate (yes/no), vigorous (yes/no), and muscle strengthening (yes/no).

### Statistical analysis

The complex survey design elements of the NHANES, including weighting, clustering, and stratification, were taken into consideration in accordance with the National Center for Health Statistics (NCHS) normative analysis guidelines. Our categorical variables were expressed as percentages, and our continuous variables were expressed as means and standard deviations. The link between ASMI, PF and BMD levels was evaluated through univariable and multivariable linear regression modeling. To further investigate the correlation between independent variables and dependent variables, we conducted multiple regressions. In the models of multivariate linear regression, an unadjusted model (Model 1) was first established, followed by an adjusted model (Model 2) that included age, gender, and race/ethnicity. A fully adjusted model (Model 3) was then calculated using variables from Models 1 and 2 plus smoking status, family income, physical activity, marital status, education level, energy, total fat and caffeine, and phosphorus intake. Moreover, we stratified the data by race/ethnicity and gender to examine the robustness of the results. In addition, non-linearity was also handled using a weighted generalized additive model and smooth curve fitting. R software (version 4.0.3; easily accessible at https://www.R-project.org) and Empower Stats (version 2.0; available at http://www.empowerstats.com) were employed for all analyses. Statistical significance was set at *P* < 0.05.

## Results

### Baseline characteristics of the participants

A total of 2165 participants were included in our analysis, with the weighted characteristics of the participants subclassified on the basis of lumbar spine BMD quartiles (Q1:0.499–0.877 g/cm^2^; Q2:0.878–0.990 g/cm^2^; Q3:0.991–1.123 g/cm^2^; and Q4:1.124–1.809 g/cm^2^), as shown in Table 1. Baseline characteristics differed significantly between the quartiles of lumbar spine BMD. In comparison with those in the lower quartiles (Q1), those in the higher quartiles (Q2–Q4) tended to be younger, male, and have higher family income, drank less, engaged in greater leisure time physical activity, and reported greater energy and caffeine intake. In addition, what is more noteworthy is that the former has higher PF and ASMI.

**Table 1:** Sociodemographic, health conditions and habits, psychical activity, anthropometric and body composition, strength, and dietary intake of person by quartile of lumbar spine BMD. NHANES, 1999–2002

Lumbar spine BMD (g/cm^2^)	Quartile 1	Quartile 2	Quartile 3	Quartile 4	*P*-value
	0.499–0.877 g/cm^2^	0.878–0.990 g/cm^2^	0.991–1.123 g/cm^2^	1.124–1.809 g/cm^2^	
*Demographic*					
Age (years)	63.96 ± 10.10	61.28 ± 9.09	59.84 ± 8.85	61.53 ± 9.70	**< 0.0001**
Gender (%)					**< 0.0001**
Male	39.37	44.57	54.1	58.34	
Female	60.63	55.43	45.9	41.66	
Race/ethnicity (%)					**< 0.0001**
Non-Hispanic White	76.60	82.12	81.62	82.17	
Non-Hispanic Black	5.34	4.75	7.97	12.91	
Mexican American	5.09	2.98	2.65	2.36	
Other race/ethnicity	12.97	10.15	7.76	2.56	
Level of education (%)					**0.0003**
Under high school graduate	54.86	50.59	43.03	41.86	
Some college or over	44.98	49.41	56.97	58.08	
Missing	0.16	–	–	0.06	
Annual family income (%)					**< 0.0001**
$0–19,999	27.44	22.52	18.94	15.53	
Over $20,000	42.15	37.27	43.89	41.54	
Missing	30.41	40.21	37.18	42.93	
Marital status (%)					**< 0.0001**
Separated/divorced/widowed/never married	33.07	27.44	22.21	23.80	
Married/living with partner	59.70	66.97	72.00	73.05	
Missing/refused/don’t know	7.23	5.59	5.79	3.15	
*Health conditions and habits*					
Hypertension (%)					**0.0123**
Yes	35.43	37.62	35.23	43.91	
No	64.57	62.38	64.77	56.09	
Arthritis (%)					0.0508
Yes	20.50	21.49	22.32	27.93	
No	47.73	44.71	42.19	40.33	
Missing	31.77	33.80	35.49	31.73	
Smoking behavior (%)					**0.0100**
Yes	18.68	19.11	16.22	13.94	
No	35.71	37.46	34.50	44.11	
Missing	45.61	43.43	49.28	41.96	
Drinking (%)					**< 0.0001**
Yes	21.77	16.53	16.54	15.23	
No	18.51	13.86	14.00	8.96	
Missing	59.73	69.61	69.46	75.81	
*Psychical activity*					
Moderate PA in past 30 days					0.449
Yes	46.69	47.35	51.05	47.47	
No	53.31	52.65	48.95	52.53	
Vigorous PA in past 30 days					**0.0006**
Yes	20.57	25.88	28.89	31.76	
No	79.43	74.12	71.11	68.24	
Strengthen PA in past 30 days					**0.0300**
Yes	18.08	15.85	20.69	22.45	
No	81.92	84.15	79.31	77.55	
*Anthropometric and body composition*					
Weight (kg)	70.24 ± 14.07	78.92 ± 17.24	82.39 ± 16.42	85.41 ± 18.41	**< 0.0001**
Height (m)	1.64 ± 0.10	1.67 ± 0.10	1.69 ± 0.10	1.71 ± 0.09	**< 0.0001**
Body mass index (kg/m^2^)	26.09 ± 4.57	28.07 ± 5.16	28.72 ± 5.32	29.27 ± 5.70	**< 0.0001**
Appendicular skeletal muscle mass (kg)	18.46 ± 4.92	20.52 ± 5.63	22.08 ± 5.47	22.92 ± 5.76	**< 0.0001**
Appendicular skeletal muscle index (kg/m^2^)	6.77 ± 1.26	7.22 ± 1.40	7.60 ± 1.35	7.77 ± 1.45	**< 0.0001**
*Strength*					
Peak force (kN)	0.33 ± 0.12	0.36 ± 0.12	0.40 ± 0.13	0.40 ± 0.13	**< 0.0001**
Time to peak force (seconds)	1.17 ± 0.73	1.16 ± 0.75	1.10 ± 0.56	1.08 ± 0.63	0.0925
Peak force velocity (degree/second)	53.70 ± 18.16	54.37 ± 17.22	55.24 ± 16.49	55.09 ± 16.72	0.4456
*Dietary intake*					
Energy (kcal/day)	1841.16 ± 923.64	1996.65 ± 905.51	2055.10 ± 919.63	2079.41 ± 919.68	**0.0002**
Carbohydrate (g/day)	235.46 ± 113.39	247.85 ± 125.61	251.57 ± 114.79	248.21 ± 114.51	0.1414
Total fat (g/day)	67.43 ± 47.36	76.03 ± 43.33	78.04 ± 45.77	79.25 ± 43.43	**0.0001**
Dietary fiber (g/day)	16.17 ± 10.33	15.82 ± 10.11	16.69 ± 10.76	16.06 ± 9.54	0.5116
Caffeine (mg/day)	199.22 ± 222.13	251.79 ± 310.44	193.66 ± 204.75	209.18 ± 206.38	**0.0001**
*Biochemical parameters*					
Albumin (g/dL)	4.33 ± 0.27	4.32 ± 0.28	4.31 ± 0.27	4.34 ± 0.31	0.4957
Total calcium (mg/dL)	9.48 ± 0.40	9.46 ± 0.41	9.42 ± 0.39	9.43 ± 0.38	0.1123
Phosphorus (mg/dL)	1.17 ± 0.17	1.13 ± 0.16	1.13 ± 0.16	1.13 ± 0.17	**< 0.0001**
Potassium (mmol/L)	4.13 ± 0.35	4.12 ± 0.35	4.13 ± 0.35	4.14 ± 0.36	0.6693

Data are described as mean ± standard error or percentage (confidence interval). Bold means that the *p*-value is statistically significant

### The relationship between BMD and PF and ASMI

An analysis of the correlation between BMD and PF and ASMI was conducted using a multiple linear regression model.

**Fig. 2:**
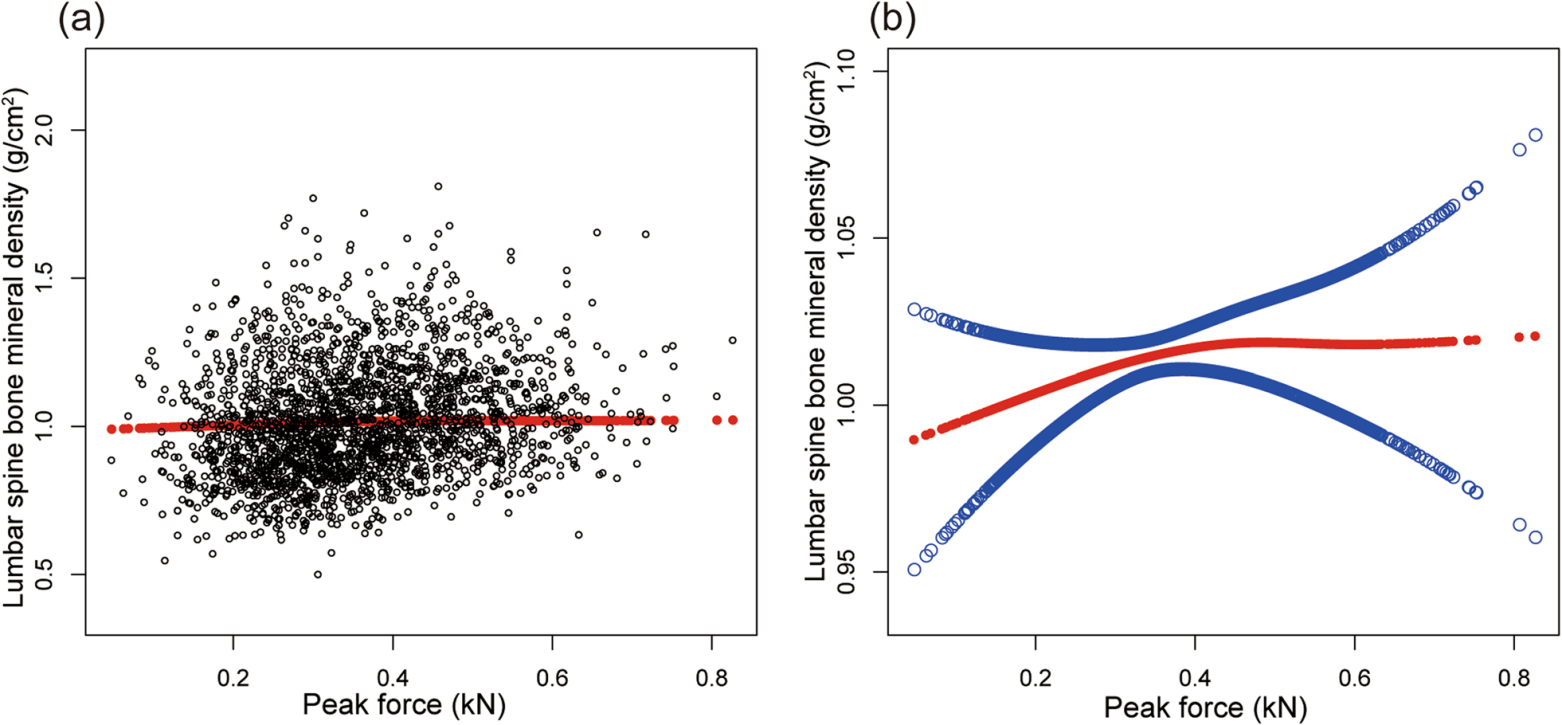
Association between peak force and lumbar spine BMD. **a** Each black point represents a sample. **b** Solid red line represents the smooth curve fit between variables. Blue bands represent the 95% of confidence interval from the fit. Adjusted for gender, age, race-ethnicity, level of education, physical activity, smoking behavior, annual family income, marital status, energy, total fat and caffeine intake and phosphorus

**Fig. 3:**
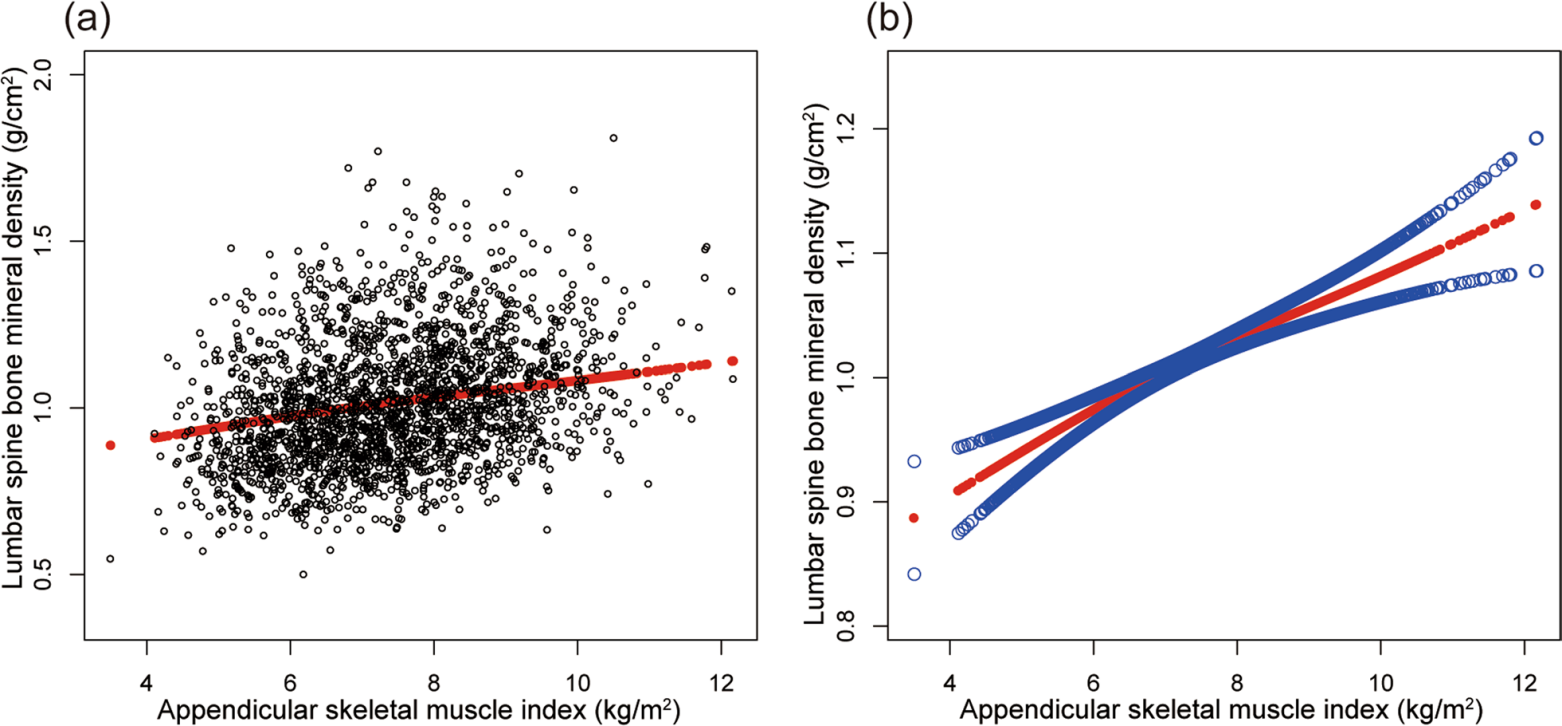
Association between ASMI and lumbar spine BMD. **a** Each black point represents a sample. **b** Solid red line represents the smooth curve fit between variables. Blue bands represent the 95% of confidence interval from the fit. Adjusted for gender, age, race-ethnicity, level of education, physical activity, smoking behavior, annual family income, marital status, energy, total fat and caffeine intake and phosphorus

**Fig. 4:**
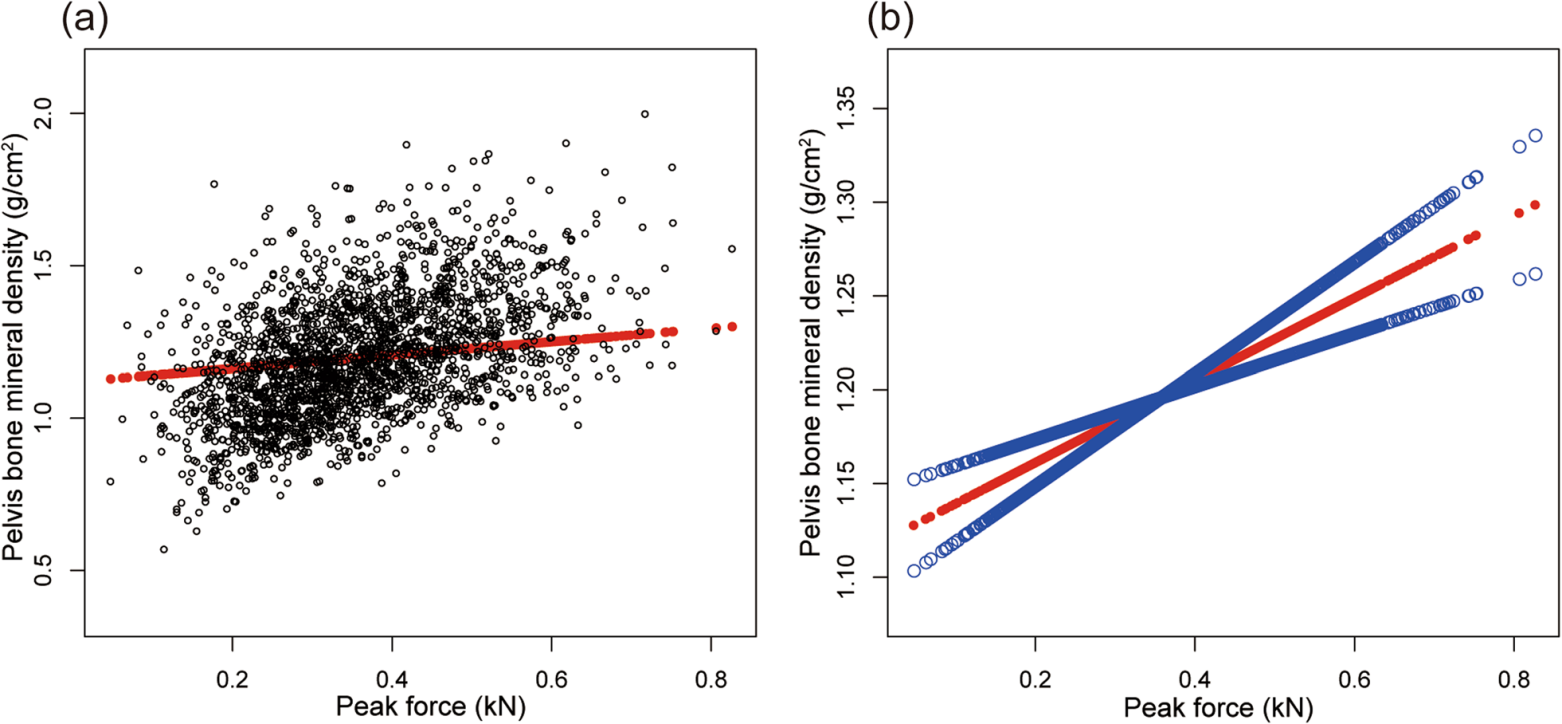
Association between peak force and pelvis BMD. **a** Each black point represents a sample. **b** Solid red line represents the smooth curve fit between variables. Blue bands represent the 95% of confidence interval from the fit. Adjusted for gender, age, race-ethnicity, level of education, physical activity, smoking behavior, annual family income, marital status, energy, total fat and caffeine intake and phosphorus

**Fig. 5:**
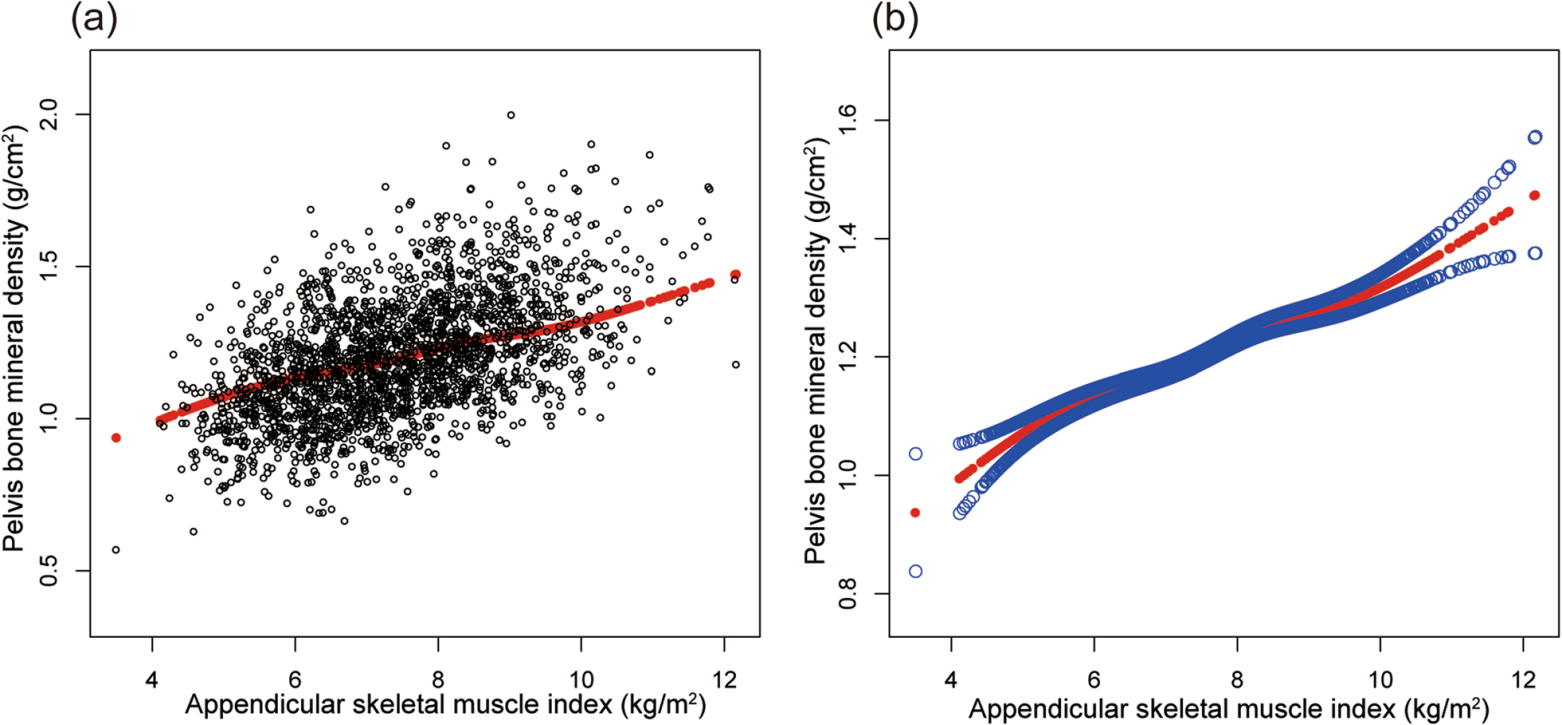
Association between ASMI and pelvis BMD. **a** Each black point represents a sample. **b** Solid red line represents the smooth curve fit between variables. Blue bands represent the 95% of confidence interval from the fit. Adjusted for gender, age, race-ethnicity, level of education, physical activity, smoking behavior, annual family income, marital status, energy, total fat and caffeine intake and phosphorus

**Fig. 6:**
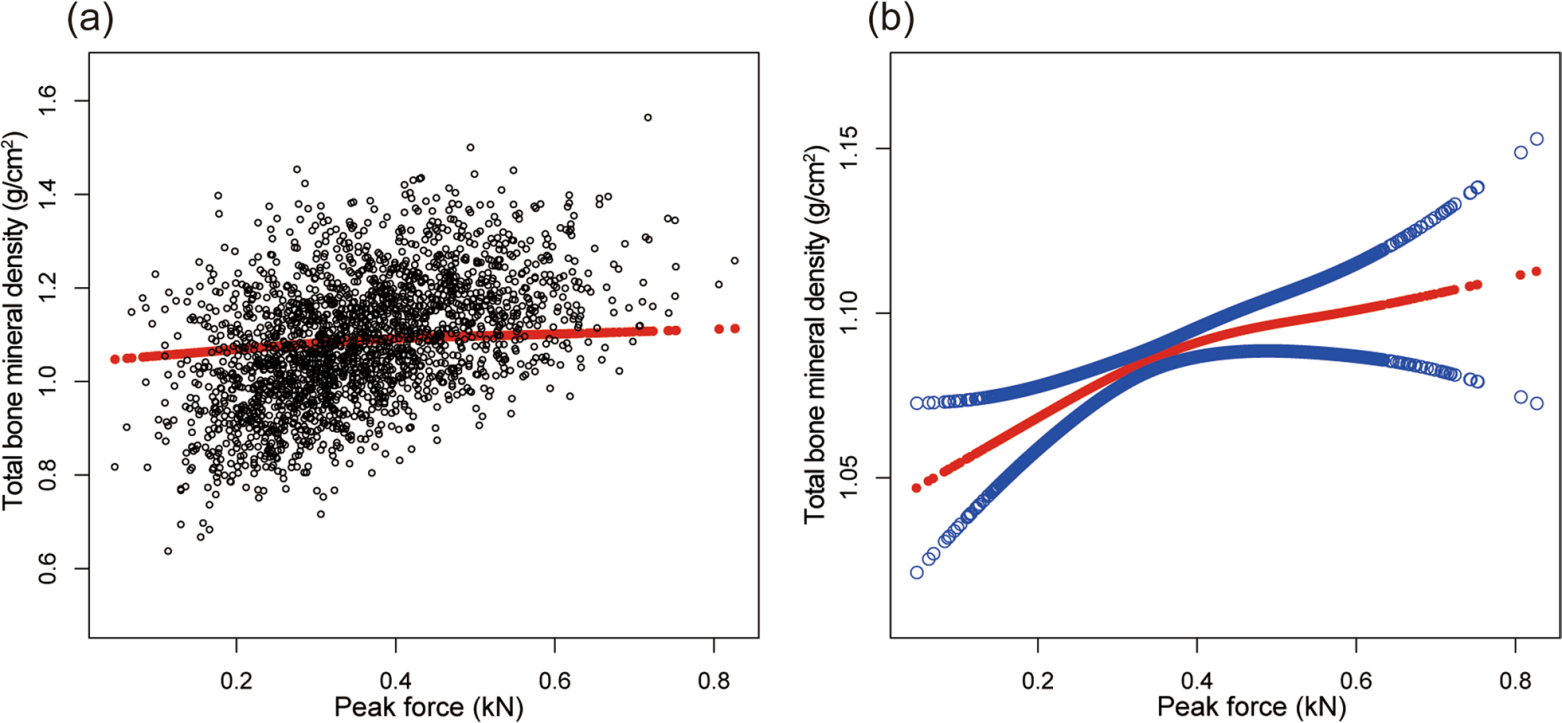
Association between peak force and total BMD. **a** Each black point represents a sample. **b** Solid red line represents the smooth curve fit between variables. Blue bands represent the 95% of confidence interval from the fit. Adjusted for gender, age, race-ethnicity, level of education, physical activity, smoking behavior, annual family income, marital status, energy, total fat and caffeine intake and phosphorus

**Fig. 7:**
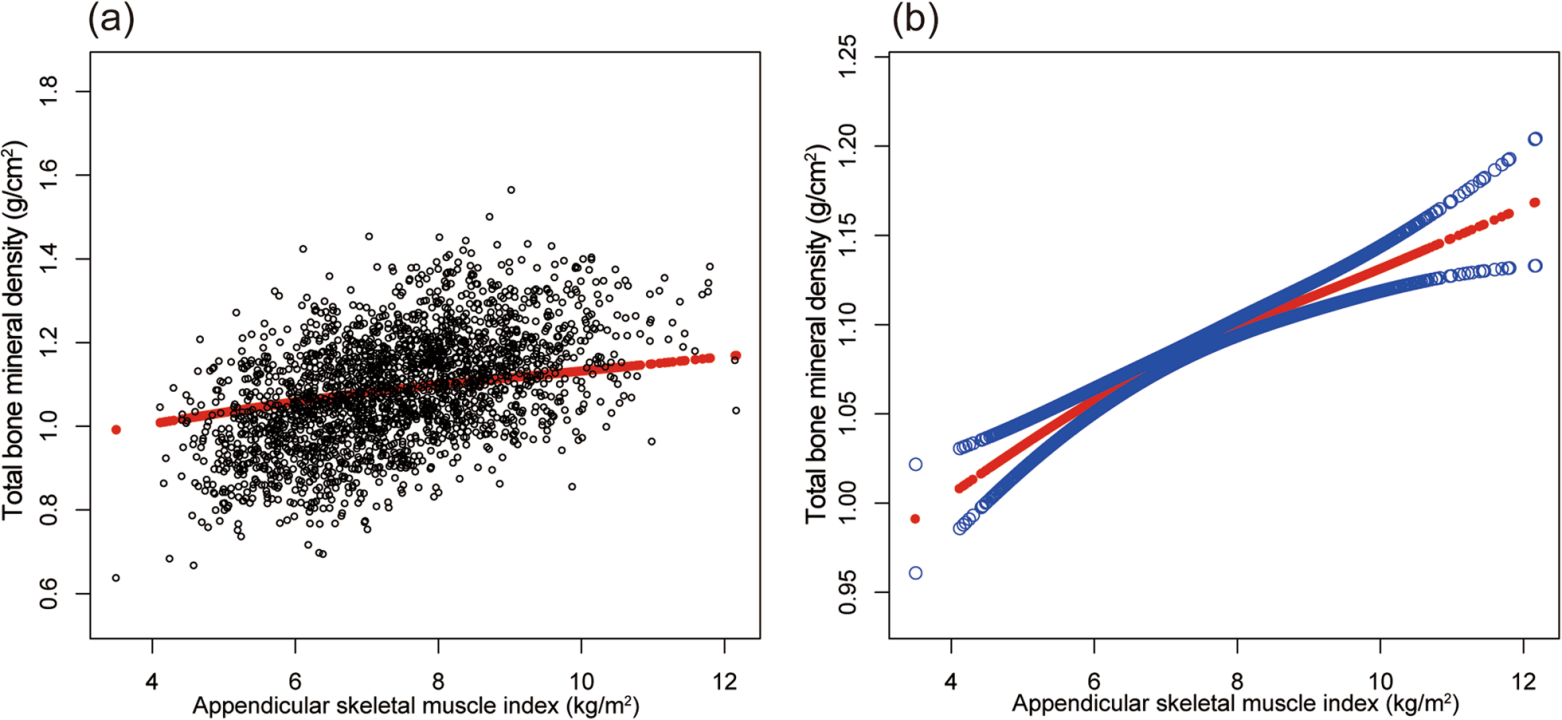
Association between ASMI and total BMD. **a** Each black point represents a sample. **b** Solid red line represents the smooth curve fit between variables. Blue bands represent the 95% of confidence interval from the fit. Adjusted for gender, age, race-ethnicity, level of education, physical activity, smoking behavior, annual family income, marital status, energy, total fat and caffeine intake and phosphorus

Table 2 shows the results of all three models. Our findings illustrate a significant correlation between muscle strength and BMD (*β* = 0.263, 95% CI 0.207–0.319,* P* < 0.00001). We also identified a significant positive relationship between lumbar spine BMD and PF (*β* = 0.167, 95% CI 0.084–0.249, *P* = 0.00008). We also found a significant association between PF and total BMD and pelvis BMD in the fully adjusted models. For muscle mass, Table 2 illustrates a positive association between ASMI and BMD (*β* = 0.033, 95% CI: 0.028–0.038, *P* < 0.00001). This association remains significant following adjustment (*β* = 0.029, 95% CI: 0.022–0.036, *P* < 0.00001). There were strong positive relationships between ASMI and total BMD, pelvis BMD across all fully adjusted models. The same trend was observed across quartiles in a sensitivity analysis, analyzing lumbar spine BMD categorical variables (quartiles), and the *P* value for the trend was 0.0001. Furthermore, we defined the nonlinear relationship between PF, ASMI, and BMD using smooth curve fitting and a generalized additive model (Figs. 2, 3, 4, 5, 6 and 7).

**Table 2:** Associations between PF, ASMI, and BMD in different models

	Model 1 *β* (95% CI, *P*)	Model 2 *β* (95% CI, *P*)	Model 3 *β* (95% CI,* P*)
*Lumbar spine BMD* (g/cm^2^)			
ASMI (kg/m^2^)	0.033 (0.028, 0.038) < 0.00001	0.032 (0.025, 0.038) < 0.00001	0.029 (0.022, 0.036) < 0.00001
PF (kN)	0.263 (0.207, 0.319) < 0.00001	0.199 (0.118, 0.280) < 0.00001	0.167 (0.084, 0.249) 0.00008
*Total BMD* (g/cm^2^)			
ASMI (kg/m^2^)	0.044 (0.040, 0.047) < 0.00001	0.022 (0.018, 0.026) < 0.00001	0.019 (0.015, 0.023) < 0.00001
PF (kN)	0.481 (0.445, 0.518) < 0.00001	0.187 (0.136, 0.237) < 0.00001	0.154 (0.104, 0.205) < 0.00001
*Pelvis BMD* (g/cm^2^)			
ASMI (kg/m^2^)	0.067 (0.062, 0.072) < 0.00001	0.054 (0.048, 0.061) < 0.00001	0.050 (0.044, 0.057) < 0.00001
PF (kN)	0.690 (0.635, 0.746) < 0.00001	0.413 (0.335, 0.490) < 0.00001	0.386 (0.308, 0.464) < 0.00001

Linear regression models:

Model 1: no covariates were adjusted

Model 2 was adjusted for demographic factors, including gender, age and race/ethnicity

Model 3 was adjusted for gender, age, race/ethnicity, level of education, physical activity, smoking behavior, annual family income, marital status, energy, total fat and caffeine intake and phosphorus

### Subgroup analyses stratified by gender and race/ethnicity

We stratified analyses by major covariates known to affect ASMI and PF to ensure that our findings are robust to the effects of potential confounding. Table 3 illustrates a subgroup analysis stratified by gender. Following adjustment, PF was positively correlated with lumbar spine BMD in both males (*β* = 0.210, 95% CI: 0.107–0.312, *P* = 0.00006) and females (*β* = 0.274, 95% CI: 0.135–0.412, *P* = 0.00011). Our findings indicate a significant relationship between ASMI and lumbar BMD in both genders. Furthermore, total BMD and pelvis BMD were clearly associated with PF and ASMI in both males and females.

**Table 3:** Subgroup analyses stratified by gender and race/ethnicity

	Model 1 *β* (95% CI, *P*)	Model 2 *β* (95% CI, *P*)	Model 3 *β* (95% CI, *P*)
*ASMI* (kg/m^2^) *(Quartile)*			
Q1	Reference	Reference	Reference
Q2	0.052 (0.032, 0.072) < 0.00001	0.051 (0.031, 0.072) < 0.00001	0.048 (0.027, 0.068) < 0.00001
Q3	0.082 (0.062, 0.102) < 0.00001	0.076 (0.053, 0.099) < 0.00001	0.069 (0.046, 0.092) < 0.00001
Q4	0.121 (0.102, 0.141) < 0.00001	0.112 (0.087, 0.137) < 0.00001	0.100 (0.074, 0.126) < 0.00001
*P* for trend	< 0.0001	< 0.0001	< 0.0001
*Peak force* (kN) (*Quartile*)			
Q1	Reference	Reference	Reference
Q2	0.037 (0.015, 0.059) 0.00081	0.035 (0.013, 0.058) 0.00192	0.031 (0.009, 0.054) 0.00702
Q3	0.064 (0.043, 0.085) < 0.00001	0.055 (0.030, 0.079) < 0.00001	0.045 (0.021, 0.070) 0.00029
Q4	0.095 (0.074, 0.115) < 0.00001	0.074 (0.046, 0.102) < 0.00001	0.063 (0.034, 0.092) 0.00002
*P* for trend	< 0.0001	< 0.0001	< 0.0001
**Lumbar spine bone mineral density**			
*Subgroup analysis stratified by gender*			
ASMI			
Male	0.032 (0.023, 0.041) < 0.00001	0.040 (0.031, 0.049) < 0.00001	0.040 (0.030, 0.050) < 0.00001
Female	0.037 (0.028, 0.045) < 0.00001	0.029 (0.020, 0.038) < 0.00001	0.031 (0.021, 0.040) < 0.00001
Peak force (kN)			
Male	0.051 (− 0.034, 0.136) 0.24027	0.228 (0.130, 0.327) < 0.00001	0.210 (0.107, 0.312) 0.00006
Female	0.521 (0.403, 0.638) < 0.00001	0.315 (0.178, 0.452) < 0.00001	0.274 (0.135, 0.412) 0.00011
*Subgroup analysis stratified by race*			
ASMI			
Non-Hispanic White	0.031 (0.025, 0.038) < 0.00001	0.035 (0.026, 0.044) < 0.00001	0.033 (0.024, 0.043) < 0.00001
Non-Hispanic Black	0.039 (0.026, 0.052) < 0.00001	0.031 (0.016, 0.046) 0.00008	0.032 (0.017, 0.048) 0.00008
Mexican American	0.030 (0.019, 0.042) < 0.00001	0.022 (0.006, 0.039) 0.00729	0.015 (− 0.001, 0.031) 0.07197
Other race/ethnicity	0.029 (0.013, 0.045) 0.00068	0.021 (0.002, 0.039) 0.03289	0.017 (− 0.003, 0.037) 0.09786
Peak force (kN)			
Non-Hispanic White	0.241 (0.169, 0.313) < 0.00001	0.212 (0.103, 0.320) 0.00013	0.163 (0.053, 0.273) 0.00378
Non-Hispanic Black	0.355 (0.182, 0.528) 0.00007	0.179 (− 0.029, 0.387) 0.09178	0.128 (− 0.088, 0.343) 0.24604
Mexican American	0.296 (0.167, 0.426) < 0.00001	0.158 (− 0.025, 0.342) 0.09206	0.104 (− 0.079, 0.287) 0.26537
Other race/ethnicity	0.327 (0.146, 0.509) 0.00054	0.210 (− 0.034, 0.455) 0.09322	0.170 (− 0.088, 0.428) 0.19864
**Pelvis bone mineral density**			
*Subgroup analysis stratified by gender*			
ASMI			
Male	0.082 (0.073, 0.091) < 0.00001	0.073 (0.064, 0.083) < 0.00001	0.070 (0.061, 0.080) < 0.00001
Female	0.055 (0.046, 0.064) < 0.00001	0.039 (0.030, 0.047) < 0.00001	0.038 (0.030, 0.047) < 0.00001
Peak force (kN)			
Male	0.539 (0.453, 0.625) < 0.00001	0.442 (0.341, 0.543) < 0.00001	0.403 (0.299, 0.507) < 0.00001
Female	0.891 (0.778, 1.004) < 0.00001	0.446 (0.321, 0.571) < 0.00001	0.419 (0.292, 0.546) < 0.00001
*Subgroup analysis stratified by race*			
ASMI			
Non-Hispanic White	0.068 (0.062, 0.075) < 0.00001	0.058 (0.050, 0.066) < 0.00001	0.055 (0.046, 0.063) < 0.00001
Non-Hispanic Black	0.067 (0.055, 0.080) < 0.00001	0.057 (0.044, 0.071) < 0.00001	0.055 (0.041, 0.069) < 0.00001
Mexican American	0.066 (0.054, 0.078) < 0.00001	0.059 (0.043, 0.074) < 0.00001	0.051 (0.035, 0.066) < 0.00001
Other race/ethnicity	0.040 (0.021, 0.058) 0.00004	0.022 (0.003, 0.042) 0.02748	0.021 (0.000, 0.041) 0.05092
Peak force (kN)			
Non-Hispanic White	0.696 (0.626, 0.767) < 0.00001	0.421 (0.318, 0.524) < 0.00001	0.387 (0.282, 0.492) < 0.00001
Non-Hispanic Black	0.686 (0.516, 0.856) < 0.00001	0.412 (0.214, 0.610) 0.00006	0.352 (0.151, 0.553) 0.00069
Mexican American	0.719 (0.588, 0.851) < 0.00001	0.474 (0.293, 0.656) < 0.00001	0.402 (0.225, 0.580) 0.00001
Other race/ethnicity	0.568 (0.370, 0.767) < 0.00001	0.317 (0.063, 0.570) 0.01571	0.249 (− 0.014, 0.512) 0.06590
**Total bone mineral density (g/cm**^**2**^**)**			
*Subgroup analysis stratified by gender*			
ASMI			
Male	0.032 (0.026, 0.037) < 0.00001	0.028 (0.023, 0.034) < 0.00001	0.027 (0.021, 0.032) < 0.00001
Female	0.029 (0.023, 0.035) < 0.00001	0.018 (0.012, 0.024) < 0.00001	0.019 (0.013, 0.025) < 0.00001
Peak force (kN)			
Male	0.232 (0.180, 0.284) < 0.00001	0.223 (0.162, 0.284) < 0.00001	0.194 (0.131, 0.256) < 0.00001
Female	0.535 (0.457, 0.613) < 0.00001	0.218 (0.132, 0.303) < 0.00001	0.184 (0.098, 0.270) 0.00003
*Subgroup analysis stratified by race*			
ASMI			
Non-Hispanic White	0.044 (0.039, 0.048) < 0.00001	0.022 (0.016, 0.028) < 0.00001	0.020 (0.014, 0.026) < 0.00001
Non-Hispanic Black	0.043 (0.034, 0.052) < 0.00001	0.025 (0.016, 0.034) < 0.00001	0.026 (0.016, 0.035) < 0.00001
Mexican American	0.044 (0.037, 0.051) < 0.00001	0.026 (0.016, 0.036) < 0.00001	0.021 (0.011, 0.031) 0.00008
Other race/ethnicity	0.035 (0.024, 0.047) < 0.00001	0.017 (0.004, 0.029) 0.00917	0.014 (0.001, 0.028) 0.03134
Peak force (kN)			
Non-Hispanic White	0.480 (0.433, 0.527) < 0.00001	0.194 (0.127, 0.261) < 0.00001	0.153 (0.086, 0.220) < 0.00001
Non-Hispanic Black	0.456 (0.340, 0.571) < 0.00001	0.132 (0.006, 0.258) 0.04145	0.075 (− 0.057, 0.208) 0.26667
Mexican American	0.496 (0.413, 0.579) < 0.00001	0.279 (0.165, 0.393) < 0.00001	0.223 (0.109, 0.337) 0.00014
Other race/ethnicity	0.442 (0.313, 0.571) < 0.00001	0.172 (0.010, 0.334) 0.03914	0.161 (− 0.008, 0.330) 0.06422

Model 1: no covariates were adjusted

Model 2: age, gender and race/ethnicity were adjusted

Model 3: gender, age, race/ethnicity, level of education, physical activity, smoking behavior, annual family income, marital status, energy, total fat and caffeine intake and phosphorus

In a race–ethnicity subgroup analysis, following adjustment, PF was positively and significantly associated with lumbar spine BMD in non-Hispanic whites (*β* = 0.033, 95% CI 0.024–0.043, *P* < 0.00001); however, this association was not significant following adjustment in Mexican Americans (*β* = 0.104, 95% CI − 0.079 to 0.287, *P* = 0.26357), non-Hispanic black (*β* = 0.128, 95% CI − 0.088 to 0.343, *P* = 0.24604), and other race-ethnicities (*β* = 0.170, 95% CI − 0.088 to 0.428, *P* = 0.19864). The positive association between ASMI and lumbar spine BMD was significant in non-Hispanic white (*β* = 0.033, 95% CI 0.024–0.043, *P* < 0.00001), and non-Hispanic blacks (*β* = 0.032, 95% CI 0.017–0.048,* P* = 0.00008); however, this association was not significant following adjustment in Mexican Americans (*β* = 0.015, 95% CI − 0.001 to 0.031, *P* = 0.07197) and other race/ethnicities (*β* = 0.017, 95% CI − 0.003 to 0.037, *P* = 0.09786). Additionally, after fully adjusting for potential confounding factors, PF and ASMI were significantly linked with pelvis BMD among non-Hispanic white, non-Hispanic black, and Mexican Americans but not in other race-ethnicities. As seen in the Model 3, we also observed that ASMI was significantly associated with total BMD across all race–ethnicities. However, PF was only moderately related with total BMD among non-Hispanic white (*β* = 0.153, 95% CI 0.086–0.220, *P* < 0.00001) and Mexican Americans (*β* = 0.223, 95% CI 0.109–0.337, *P* = 0.00014).

## Discussion

We utilized data from the NHANES 1999–2002 for this cross-sectional study. The results of the current study suggest that muscle mass and strength are significantly and positively associated with BMD both in univariate and multivariate linear regression analyses. The strong association between muscle mass and strength and BMD persisted after controlling for multiple covariates.

Previous studies have indicated that muscle strength is closely related to BMD [28]. Similarly, Elhakeem et al. [29] discovered that hip BMD in postmenopausal women was independently associated with peak lower extremity muscular strength; an analysis of 979 postmenopausal women from Finland (mean age 68.1 years) in a population-based study illustrated that both body composition and muscle strength had a substantial impact on bone density [28]; furthermore, research by Seabra et al. [30] demonstrated that in teenagers, lower limb muscular strength was related to BMD and bone mineral content (BMC) across the board; a connection between older people’s muscle mass, strength, and bone mass was shown to be gender-specific in a prospective community-based study [31]. In contrast, our findings indicated that muscular strength and BMD are positively associated and do not differ based on gender. Two considerations might account for the contrast: first, variations in research design, muscle-measuring tools, or statistical correction factors; second, variations in sample sizes between investigations.

By employing ASMI, we later examined the connection between muscle mass and BMD and observed that there was a strong significant relationship between the two variables. This is consistent with the findings of Segal et al. [32], who revealed that muscle mass was positively associated with hip BMD. According to a cross-sectional investigation, the incidence of spinal compression fractures may be substantially related to lower limb muscle mass and grip strength [33]. In addition, compared with the study of Qin et al. [10], our study selected a wider range of people, no longer limited to those under 59 years old. Moreover, for BMD, this study selected a greater amount of regional BMD data, which should ensure that the conclusions of this paper are more rigorous. Compared with some previous studies with small sample-size studies, we utilized nationally representative NHANES data, which improved the external validity. Thus, this survey will give convincing support for the use of ASMI and muscular strength testing for osteopenia and osteoporosis screening methods in the USA.

There has been a struggle among researchers to understand how bone health and muscle are linked. In Bone’s mechanostat theory, mechanical strain applied to bone determines bone remodeling, and bones adapt to static and dynamic forces generated by muscle contractions [34]. As muscles become stronger, they may exert greater stress on their bones, putting them under load and encouraging bone formation and retention, which contributes to bone mass [35]. Skeletal muscle mass increases with an increase in mechanical load, whereas skeletal muscle mass decreases with a decrease in mechanical load [36]. There is evidence that skeletal muscle-derived mechanical loading plays an important role in bone development and maintenance [37, 38]. Besides being mechanically active, skeletal muscle can also function endocrinely to maintain skeletal homeostasis. It is believed that skeletal muscle is an endocrine organ that releases a set of cytokines and a protein called myokines whenever muscles contract. Muscle myokines function autocrinely by controlling muscle metabolism, while bone, fat, brain, and liver myokines function paracrinely [39]. A mouse model of limb muscle removal affects bone growth and mineralization; however, mice receiving minced skeletal muscle, non-limb SKM, or cardiac muscle were able to form cartilage and bone nodules [40]. Animals with fractures where a skeletal muscle segment is excised heal better (and even increase their bone synthesis) when high molecular weight molecules derived from the muscle are infused into the injured area [41, 42]. The skeletal muscle releases actin directly to regulate the interaction between the muscle and the bones. In addition, the underlying mechanisms of muscle effects on BMD, including mechanical and metabolic aspects, still require further investigation.

A gender-based subgroup analysis was conducted to determine if gender affected bone metabolism. In both genders, PF, ASMI, and BMD correlated significantly and positively. The association remained significant after adjusting for multiple confounding factors, suggesting that the relationship was independent of gender.

However, analyses stratified by race/ethnicity in our group showed that ASMI and PF were consistently positively associated with BMD in non-Hispanic whites, whereas associations became less significant in other racial/ethnic groups. And this means that race/ethnicity can influence the association of ASMI and PF with BMD in this study. A cross-sectional study of 1190 community-dwelling men showed that greater bone strength among black American men than among their white counterparts and may indicate elevated fracture risk among older Hispanic American subpopulations [43]. The same is true for women; studies from NHANES III reported higher femoral neck BMD and shorter hip axis length in Mexican American women compared to non-Hispanic white women in the USA [44]. Consistent with the previous study, non-Hispanic whites have been reported to have significantly lower BMD and higher fracture rates compared with non-Hispanic blacks [45–47]. Moreover, Noel et al. [48] demonstrated differences in BMD and osteoporosis among major racial and ethnic groups, highlighting the need to study individual groups by origin or background. It is worth noting that research by Berenson et al. [49] discovered differences in genetic risk factors, obesity status, alcohol consumption, and other factors across racial/ethnic groups, which may provide a possible explanation for noted race-specific differences. Future studies with larger populations would benefit from examining associations across a wider range of race/ethnicity.

Our research has several advantages. First, it is accomplished by analysis of the NHANES data, a dependable data source for epidemiological studies. There is less likelihood of sampling bias because NHANES contains a substantial representative sample of the whole US population. Moreover, questionnaires and laboratory data sets offer comprehensive demographic, lifestyle, dietary, and medical information. We can better manage possible confounders in multiple regression models with the use of this data. We also have more ability to produce meaningful results thanks to the large sample size that NHANES provides. Finally, the knee extensor peak strength test is a commonly used muscle strength measure. ASMI is easier to obtain with DXA, so both are easier to use as screening tests for low BMD.

The study has several limitations. On the one hand, as a result of the cross-sectional design of this study, causal relationships between muscle strength, mass, and BMD cannot be inferred. On the other hand, even though we included a number of covariables when performing the multivariate regression analysis, there may still be some residual confounding.

## Conclusion

We found that muscle mass and strength were significantly and positively associated with BMD in the total, pelvis, and lumbar spine in nationally representative sample of US adults, suggesting that PF and ASMI in the lower extremities may be a screening indicator for low BMD. This makes it easier to intervene early in osteoporosis risk individuals. For future research direction, it is suggested to further explore the causal relationship between muscle mass, strength, and BMD. This will be very helpful for our future research.

## Data Availability

The survey data were publicly available on the internet for data users and researchers throughout the world http://www.cdc.gov/nchs/nhanes/.

## Abbreviations

US: The United States
BMD: Bone mineral density
BMC: Bone mineral content
PF: Peak force
ASMI: Appendicular skeletal muscle index
ALM: Appendicular lean mass
ASM: Appendicular skeletal muscle mass
NHANES: The National Health and Nutrition Examination Survey
NCHS: The National Center for Health Statistics
DXA: Dual-energy X-ray absorption
PA: Physical activity
